# Contribution of the pod wall to seed grain filling in alfalfa

**DOI:** 10.1038/srep26586

**Published:** 2016-05-23

**Authors:** Hui Wang, Longyu Hou, Mingya Wang, Peisheng Mao

**Affiliations:** 1 Forage Seed Laboratory, China Agricultural University, Beijing 100193, P. R. China; 2 Beijing Key Laboratory of Grassland Science, Beijing 100193, P. R. China

## Abstract

Three genotypes of alfalfa viz. *Medicago sativa* (Zhongmu No. 1, Zhongmu No. 2) and *M. varia* (Caoyuan No. 3) grown in the filed were investigated for the contribution of pod wall and leaves by shading all pods and leaves on July 15, 20 and 25, respectively. Date was recorded for total pod weight (TPW), pod wall weight (PWW), seed weight per pod (SWP), seed number per pod (SNP) and single seed weight (SSW) of one-coil and two-coil spiral pods. TPW, SNP, PWW and SWP were reduced by shading all leaves or pods, whereas SSW was not significantly affected. The relative photosynthetic contribution of pod wall to SWP was 25.6–48.1% in three genotypes on July 15. The pod wall in one-coil spiral pods generated a greater relative contribution to the TPW and SWP than in two-coil spiral pods. In the last stage (July 25), the relative photosynthetic contribution of leaves to SWP sharply decreased, whereas the relative photosynthetic contribution of pod wall to SWP was stable in the late stage (July 20 and 25). In conclusion, the pod wall of alfalfa could carry out photosynthesis and the pod wall played an important role in pod filling at the late growth stage.

A total of 90%–95% of crop biomass is generated from photosynthesis. Generally, leaves are restrictively considered the photosynthesis and carbohydrate production organs. Nevertheless, in addition to leaves, evidence of photosynthetic activity in non-leaf organs has been found in, for example, the pods of Brassica (*Brassica napus* L.)^1^, the spikelets in rice (*Oryza sativa* L.)^2^, the awns in wheat (*Triticum aestivum* L.)^3^, the inflorescence in grapevine (*Vitis vinifera* L.)^4^, the fruit in tomatoes (*Lycopersicon esculentum* Mill.)^5^ and the capsule in cotton (*Gossypium* spp.)^6^. For legumes, many researchers have focused on the photosynthetic activity of pod wall, such as in soybeans (*Glycine max* L.)^7^,^8^, beans (*Phaseolus vulgaris* L.)^9^, peas (*Pisum sativum* L.)^10^ and chickpeas (*Cicer arietinum* L.)^11^,^12^,^13^. It is now accepted that the pod wall plays a necessary and excess role in the carbohydrate acquisition of seeds. Two distinct photosynthetic layers containing chloroplasts are present in the pod wall of peas, one capturing CO_2_ from the outside atmosphere through the stomata and the other obtaining CO_2_ released from respiring seeds^10^. Furthermore, the internal recycling of CO_2_ inside the pod contributes to maintaining seed filling under drought conditions, whereas the leaves present a low rate of photosynthesis^12^. The relative contribution of the pod wall has been measured by previous investigators, for instance, 14–24% contribution to the seed weight in chickpeas^14^ and 7.34–15.06% to the seed weight in soybeans^15^. With increasing pod size, the chlorophyll content in the pod decreases, and respiration and gross photosynthesis had the same tendency, but the rate of gross photosynthesis based on chlorophyll in the pods exhibited an inverse pattern, and the pods had greater gross photosynthetic rates than leaves in terms of chlorophyll^9^. Furthermore, the photosynthesis of the pod wall in the late stage of pod-filling was steady and contributory, even when the photosynthetic ability of the leaves trended toward decreasing^16^. Furthermore, the pod wall connects directly with seeds, which is advantageous for transferring photosynthate to seeds.

Alfalfa (*Medicago sativa* L.) is a superior leguminous forage crop that is grown worldwide due to its high hay yield potentials, excellent quality and good adaptability, but its seed yield is deemed to be of secondary importance^17^. However, for a new alfalfa cultivar, the commercial value is embodied not only in its forage production but also in its seed attributes^18^. Studying the photosynthetic activity of the pod wall may be a new approach to increasing seed yields. Unlike soybeans, chickpeas and peas, the alfalfa pod is shaped like an open spiral and has one to three and rarely four and five coils. Among alfalfa pods, the percentage of one-coil spirals and two-coil spiral pods is 54.2% and 42.6%, respectively^19^.

Research has analyzed the photosynthesis of the pod wall in some legumes^8^,^13^,^20^. However, little research has been performed on the photosynthetic contribution of the pod wall in alfalfa. This study investigated whether the pod wall of alfalfa could carry out photosynthesis and the relative contribution of the pod wall to pod weight and seed weight.

## Results

### Effect of shading treatments on pods

The results determined that the TPW of two pod types in three genotypes under shading all leaves at the same stages was lower than under shading all pods, except for the one-coil spiral pods on July 15, whereas the shaded pods could reduce the TPW compared to the control (Table 1). The TPW of the two pod types increased with shading stage from July 15 to July 25 for each genotype under shading all leaves or pods. Zhongmu No. 1 had a greater TPW in the control than Zhongmu No. 2 and Caoyuan No. 3 for both pod types.

According to these results, the PWW was differently affected by shading all leaves or pods in the three stages for both pod types of the three genotypes (Table 1). For the one-coil spiral pods, the PWW was lighter in three genotypes under shading all leaves or pods in three stages than that of the control and increased with shading stage from July 15 to July 25 for each genotype under shading all leaves or pods. In contrast, for the two-coil spiral pods, there was no significant (*P* > 0.05) difference in the PWW under shading all leaves and pods at three stages for each genotype, except for Zhongmu No. 1.

For the one-coil spiral pods, shading all leaves or pods in the three stages could significantly (*P* < 0.05) reduce the SWP in three genotypes compared to that of the control (Table 1). However, for the two-coil spiral pods, except for in Zhongmu No. 1, the SWP decreased insignificantly (*P* > 0.05) under shading all leaves or pods in both Zhongmu No. 2 and Caoyuan No. 3. This study showed that the SWP of one-coil spiral and two-coil spiral pods increased with the shading stage from July 15 to July 25 under shading all leaves or pods in Zhongmu No.1 and Zhongmu No. 2.

The SNP of both pod types in each genotype decreased from the shading of all leaves or pods in three stages compared to the control, except for the two-coil spiral pods in Zhongmu No. 2 (Table 1). The experimental data indicated that the SNP under shading all leaves or pods in the late period (July 20 and 25) for three genotypes was higher than in the early period (July 15), except for the one-coil spiral pods in Caoyuan No. 3.

From the results, there was no significant (*P* > 0.05) difference between the SSW from the shading of all leaves and pods in the three stages for two pod types in three genotypes (Table 1). A greater SSW was observed in Zhongmu No. 1 and Caoyuan No. 3 than in Zhongmu No. 2.

### The correlation of PWW and SWP with TPW and the correlation of SNP and SSW with SWP in both pod types

**Figure 1:**
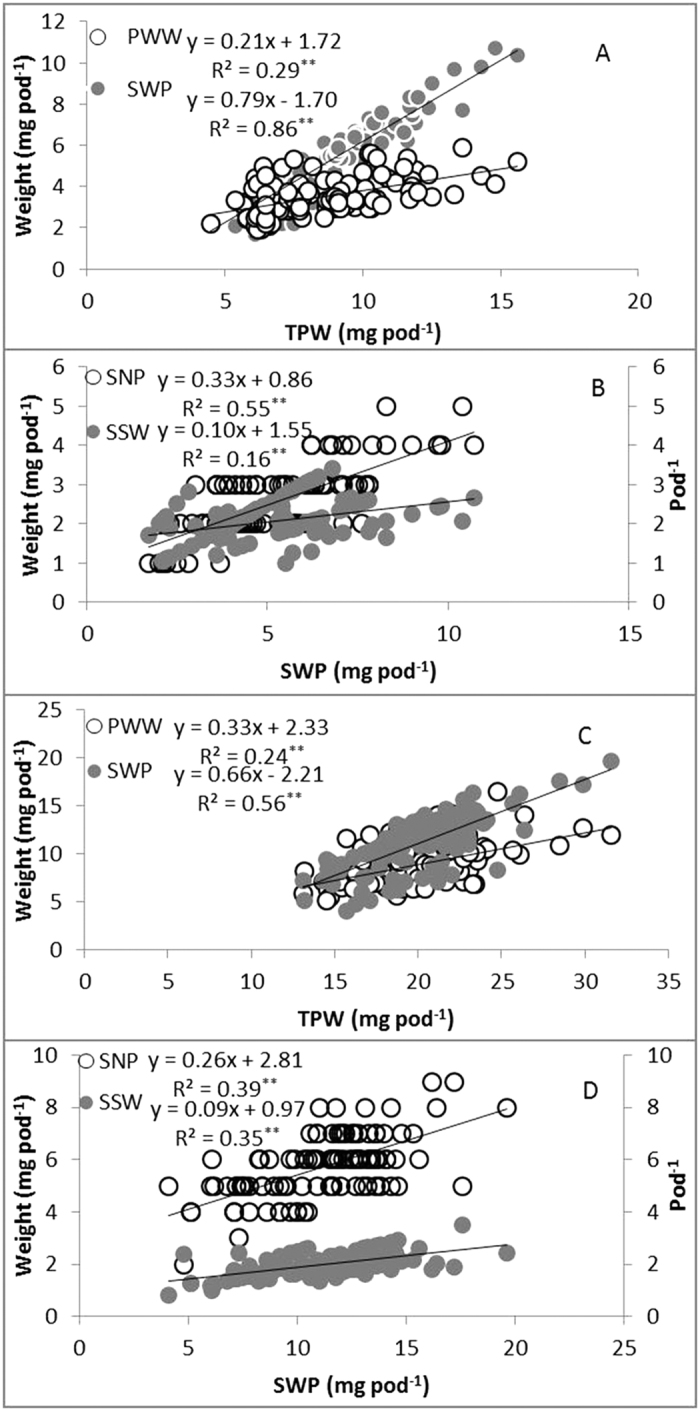
The relationships between pod wall weight per pod (PWW) and seed weight per pod (SWP) with the total pod weight per pod (TPW) and the relationship between seed number per pod (SNP) and single seed weight (SSW) with seed weight per pod (SWP). (**A**) The relationships between the PWW and SWP with TPW in the one-coil spiral pod. (**B**) The relationships between the PWW and SWP with TPW in the two-coil spiral pod. (**C**) The relationship between the SNP and SSW in the one-coil spiral pod. (**D**) The relationship between the SNP and SSW in the two-coil spiral pod. **Significantly different at the 0.05 level.

The PWW and SWP were significantly (*P* < 0.01) and linearly correlated with the TPW of both pod types (Fig. 1A,C). However, the SWP (one-coil spiral pod, R^2^ = 0.86; two-coil spiral pod, R^2^ = 0.56) could explain more proportion of variation of TPW than PWW (one-coil spiral pod, R^2^ = 0.29; two-coil spiral pod, R^2^ = 0.24). Furthermore, the significant (*P* < 0.01) relationship between the SNP or SSW and SWP occurred in one-coil pods (Fig. 1B,D). For one-coil spiral pods, 55% of the variation in the SWP could be explained by the relationship with the SNP, whereas the SSW could explain a lower proportion of variation in the SWP (R^2^ = 0.16, Fig. 1C). Nevertheless, for two-coil spiral pods, the SNP (R^2^ = 0.39) and SSW (R^2^ = 0.35) could explain a similar proportion of variation in the SWP (Fig. 1D).

### The effect of shading treatments on the percentage of PWW and SWP in the TPW

The percentage of PWW and SWP in the TPW was calculated for both pod types and the three shading stages (Table 2). Shading all the pods could significantly (*P* < 0.05) reduce the percentage of SWP in the TPW for all three genotypes. With the exception of Caoyuan No. 3, the percentage of SWP in the TPW significantly (*P* < 0.05) decreased through shading all leaves. There was no significant (*P* > 0.05) difference between the percentage of SWP in the TPW from the shading of all leaves and the shading of all pods in Zhongmu No. 1 and Zhongmu No. 2.

### The relative contribution of the leaves and pod wall to the TPW and SWP

**Figure 2:**
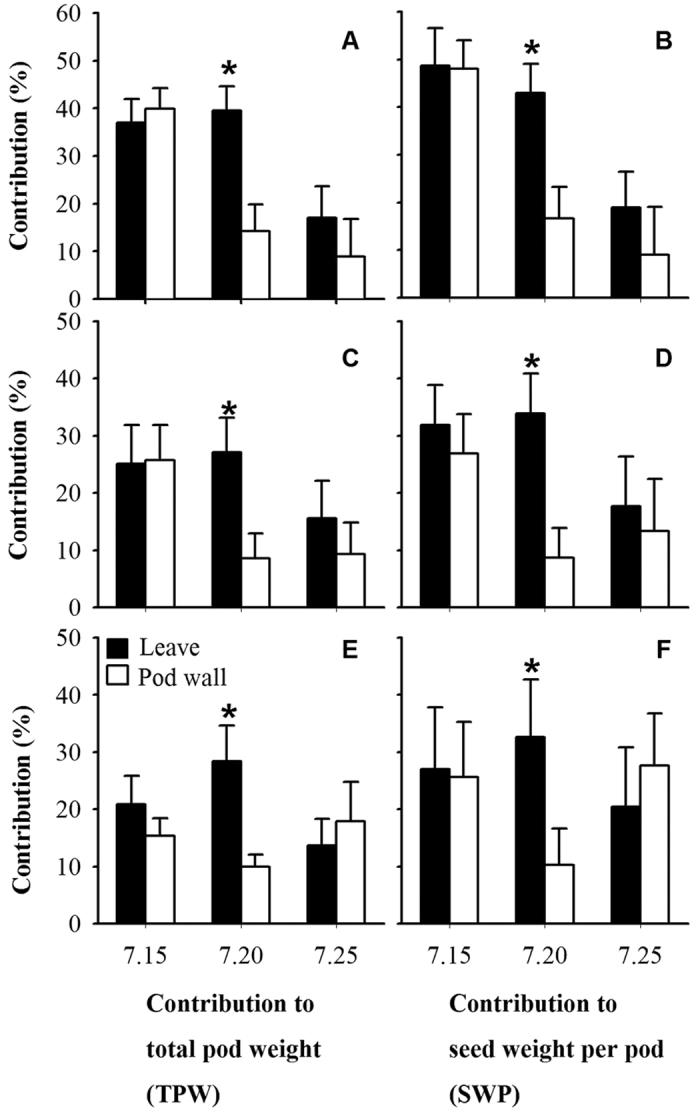
The relative contribution of the leaves and pod wall to the total pod weight (TPW) and seed weight per pod (SWP) in three cultivars. (**A**) The relative contribution of the leaves and pod wall to the TPW in Zhongmu No. 1. (**B**) The relative contribution of the leaves and pod wall to the SWP in Zhongmu No. 1. (**C**) The relative contribution of the leaves and pod wall to the TPW in Zhongmu No. 2. (**D**) The relative contribution of the leaves and pod wall to SWP in Zhongmu No. 2. (**E**) The relative contribution of the leaves and pod wall to TPW in Caoyuan No. 3. (**F**) The relative contribution of the leaves and pod wall to the SWP in Caoyuan No. 3. *P < 0.05.

The relative photosynthetic contribution of the leaves and pod wall to the TPW and SWP was assessed from the reduction in their weight from the shading of all leaves or pods in the three genotypes (Fig. 2). For a single pod, in the early (July 15) and last stages (July 25) of every genotype, the pod wall had a similar relative photosynthetic contribution to the TPW or SWP as the leaves, whereas the relative photosynthetic contribution of the leaves was significantly (*P* < 0.05) higher than the pod wall in the middle stage (July 20). In the early stage (July 15) of growth in every genotype, the leaves had the large relative contribution to the TPW and SWP, whereas the relative contribution of the leaves to the TPW and SWP decreased sharply and significantly (*P* < 0.05) in the last stage (July 25). Nevertheless, for the pod wall, the relative contribution to the TPW and SWP was steady.

## Discussion

There have been many studies about non-leaf photosynthesis^2^,^4^,^6^, but this study was the first to analyze the photosynthetic contribution of the pod wall in alfalfa. According to these research results, shading all pods of the branches for each genotype could significantly (*P* < 0.05) reduce the TPW and SWP (Table 1), which indicateed that the pod wall of alfalfa could be carrying out photosynthesis. These results were similar to the findings indicating that the seed weight per plant could be decreased by shading bolls in cotton^21^. Chloroplasts and stomata, which were crucial to photosynthesis, had been observed in the pods^10^,^22^. Furthermore, the pod had 40% as much RuBP (ribulose 1, 5 diphosphate carboxylase) and (GO) glycolate oxidase activity and over 700% as much malate dehydrogenase activity per unit area as the leaves^20^. Therefore, like other non-leaf organs, the pod wall in alfalfa underwent photosynthesis that was additive and necessary.

The TPW and SWP were significantly (*P* < 0.05) affected by shading all pods. However, the PWW was insignificantly (*P* > 0.05) influenced by shading all pods. This could be accounted for by other assimilate supply sources, particularly the stems, which might be conducive to the dry matter accumulation of the pod wall^23^. A lower proportion of variation in the TPW was explained by the relationship with PWW compared with SWP (Fig. 1A,C). Similarly, the shading treatments could not significantly (*P* > 0.05) affect SSW. According to the results, SNP could explain a greater proportion of variation in SWP than SSW (Fig. 1B). The shading treatments reduced the photosynthesis source, resulting in maintenance of individual seed weight by decreasing SNP. The seed number had a negative correlation with seed weight^24^. Furthermore, cutting off photosynthetic sources could decrease the seeds per head, while the seed weight remains steady^25^.

The relative photosynthetic contribution of the pod wall exhibited a visual index to reflect the importance of the pod wall in legumes. In the early stage (July 15), the maximum relative photosynthetic contribution of the pod wall to the SWP was observed in Zhongmu No. 1 (48.1%, Fig. 2B). The pod wall in Zhongmu No. 2 (26.9%) had a relative photosynthetic contribution to SWP similar to that in Caoyuan No. 3 (25.6%). The results indicated that there was variation in the relative photosynthetic contribution of the pod wall among the different genotypes. Similar results were reported in previous research for *Brassica*^23^ and chickpeas^14^. Reductions in SWP in fifteen genotypes of *Brassica* ranged from 40.9% to 90.1% from covering all pods with aluminum foil five days after anthesis^23^. Twelve chickpea genotypes exhibited a reduction in dry seed weight, from 0.8% to 20.8%, when all the pods were shaded with aluminum foil^14^. Furthermore, this study showed that there was a significant (*P* < 0.05) difference between the relative photosynthetic contribution of leaves in the last stage (July 25) and in the middle stage (July 20), whereas the state of the pod wall varied. Research indicated that leaves start to senesce earlier than other organs^26^. Thus, the pod wall contributed to maintaining photosynthate supply in the last period of alfalfa growth.

**Figure 3:**
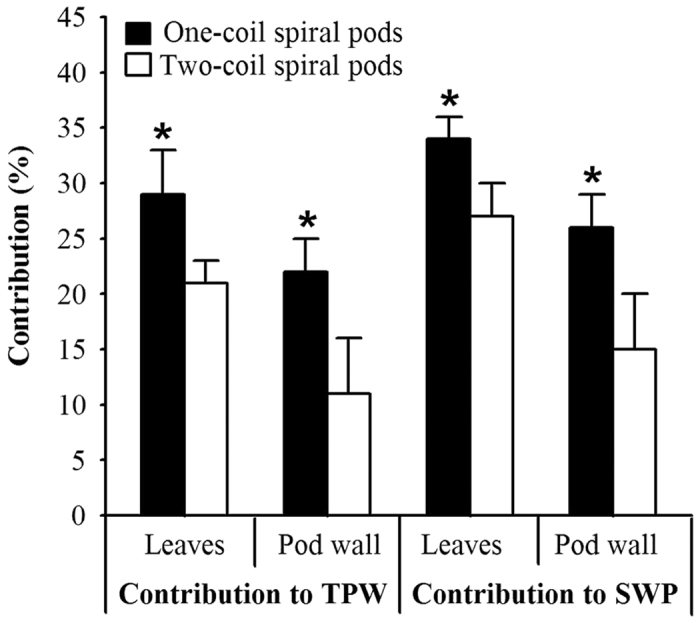
The relative contribution of the leaves and pod wall in one-coil spiral and two-coil spiral pods to the total pod weight (TPW) and the seed weight per pod (SWP). *P < 0.05.

The results of the present study indicated no significant (*P* > 0.05) difference among PWW or SWP under shading all leaves and pods in three stages in two-coil spiral pods (Table 1). Furthermore, 35% of the variation in the SWP could be explained by the relationship with SSW in the two-coil spiral pods, whereas 16% of the variation occurred in the one-coil spiral pods. The above results indicated that the two-coil spiral pods had a more stable character than the one-coil spiral pods. Furthermore, the relative contribution of the pod wall in the one-coil spiral pods to the TPW and SWP were greater than in the two-coil spiral pods (Fig. 3). The two-coil spiral pods had a greater photosynthetic surface area than the one-coil spiral pods, whereas more seed numbers were observed in the two-coil spiral pods, requiring more photosynthetic products to maintain development and growth compared to the one-coil spiral pods^27^.

## Conclusions

The present study has evaluated the TPW, SWP and relative photosynthetic contribution under shading all leaves and pods. Our results indicate that the pod wall of alfalfa provides a large photosynthetic contribution, equal to the leaves, to seed filling in the early growth stage and a steady photosynthetic product supply in the late growth stage in alfalfa. Our study provides a novel perspective of alfalfa seed production. Further research is needed to understand how to utilize the photosynthetic performance of the pod wall to increase seed yields in alfalfa.

## Methods and Materials

### Experimental site

The experiment field was located in the Etuoke Banner of the Inner Mongolia Autonomous Region of China (latitude, 39°12′N; longitude, 106°95′E; elevation, 1150 m). The soil was a gray meadow soil. The soil (0–30 cm) could be described as follows: pH, 8.89; organic matter, 9.76 g kg^−1^; alkaline-hydrolysable nitrogen, 72.4 mg kg^−1^; phosphorus, 7.28 mg kg^−1^; potassium, 76 mg kg^−1^; and calcium, 92 mg kg^−1^.

### Experimental materials

Three genotypes, belonging to two species of alfalfa, namely, *Medicago sativa* (Zhongmu No. 1 and Zhongmu No. 2) and *M. varia* (Caoyuan No. 3), were grown under field conditions following recommended agronomic practices.

### Field trials design

**Figure 4:**
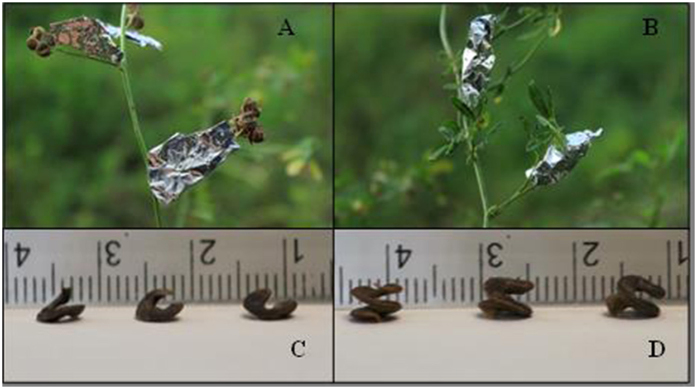
Shading treatments and two pod types. (**A**) Shading all leaves by covering with aluminum foil. (**B**) Shading all pods by covering with aluminum foil. (**C**) one-coil spiral pods. (**D**) two-coil spiral pods.

The field was established in October of 2008 and the experiment was arranged in a randomized complete block design with three replicates. The individual plot size was 36 m^2^ (4.5 m × 8 m). This trial was carried out in 2014, and when pod emergence started on July 5, thirty branches (six branches per plant  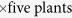) of every genotype were chosen to divide randomly and averagely into three groups on July 15, 20 and 25, around the pod-bearing, pod-filling and after the pod-filling period, respectively. Three treatments (two branches per plant for one treatment, for a total of six branches) were conducted in the above three groups until harvest, as follows: shading all leaves with aluminum foil with 1 mm holes (Fig. 4A), shading all pods using the same method as shading all leaves (Fig. 4B) and no shading (the control).

### Data collection

All the pods were harvested on August 5. Twenty one-coil spiral (Fig. 4C) and two-coil spiral pods (Fig. 4D) were picked to dry to a constant dry weight at 85 °C and the dry weight was recorded from all pods in different treatments and the control and was then divided into the pod wall and seeds, respectively. The dry weight of the pod wall per pod and the seeds per pod were weighed and the seed number per pod was counted.

Single seed weight (SSW) = seed weight per pod (SWP)/seed number per pod (SNP).

Relative contribution (%) = 100 × (control weight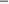 shading weight)/control weight^21^. This formula was used to calculate the relative contribution from the leaves and pod wall to the TPW and SWP.

### Statistical analyses

The tests were completed following a randomized statistical design. The data were analyzed as a three-factor experiment, using three genotypes (Zhongmu No. 1, Zhongmu No. 2, Caoyuan No. 3), seven shading treatments (the control + shading all leaves and shading all pods × three stages) and two pod types (one-coil spiral pod and two-coil spiral pod). The data were analyzed using an ANOVA and the means of each treatment were compared using a Fisher’s protected LSD test with a significance level of 0.05. These analyses were performed using SAS (version 8.0).

## Additional Information

**How to cite this article**: Wang, H. *et al.* Contribution of the pod wall to seed grain filling in alfalfa. *Sci. Rep.* **6**, 26586; doi: 10.1038/srep26586 (2016).

## Figures and Tables

**Table 1:**
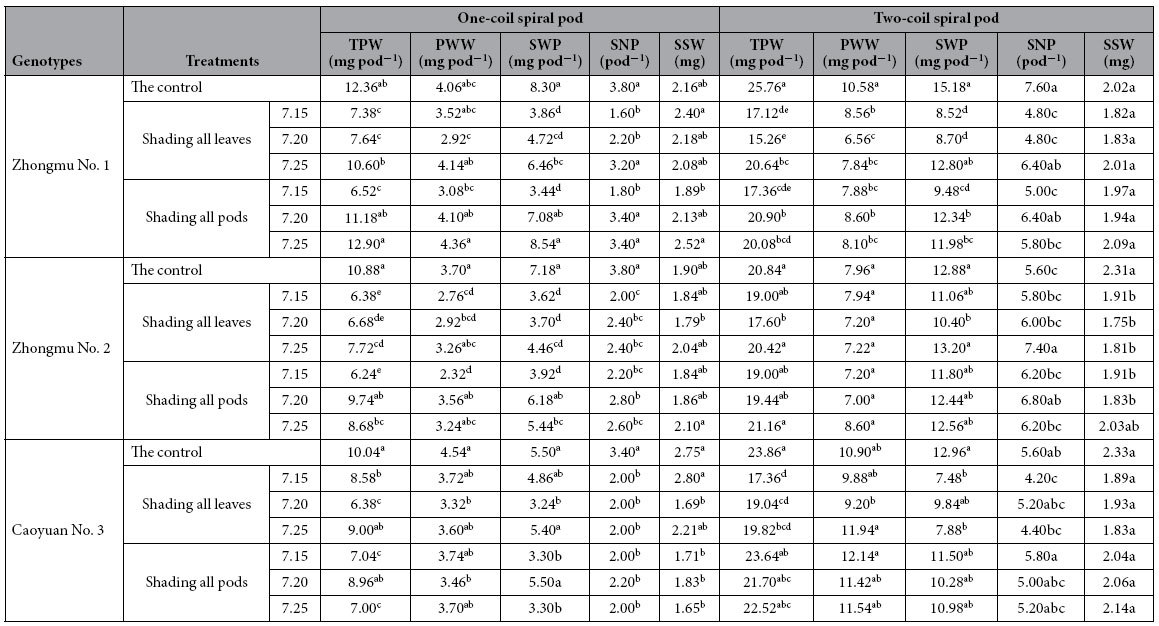
Total pod weight (TPW), pod wall weight (PWW), seed weight per pod (SWP), seed number per pod (SNP) and single seed weight (SSW) of the two pod types in three genotypes as influenced by shading all leaves or pods on July 15, 20 and 25 (LSD test, *P *< 0.05).

**Table 2:** Percent of pod wall weight (PWW) and seed weight per pod (SWP) in the total pod weight (TPW) (LSD test, *P* < 0.05).

Genotypes	Treatments	PWW / TPW (%)	SWP / TPW (%)
Zhongmu No. 1	The control	33^b^	67^a^
	Shading all leaves	41^a^	59^b^
	Shading all pods	40^a^	60^b^
Zhongmu No. 2	The control	34^b^	66^a^
	Shading all leaves	41^a^	59^b^
	Shading all pods	40^a^	60^b^
Caoyuan No. 3	The control	45^b^	55^a^
	Shading all leaves	41^b^	59^a^
	Shading all pods	53^a^	47^b^
